# Basal Ganglia Calcifications With Acute Behavioral Changes: A Case of Fahr’s Syndrome

**DOI:** 10.7759/cureus.97802

**Published:** 2025-11-25

**Authors:** Bassem Al Hariri, Areej A Ali, Areej A Hassan, Suad A Abker, Amna S Satti, Mohamed Gadkarem, Osama H Mohammed, Abdulqadir J Nashwan

**Affiliations:** 1 Internal Medicine Department, Hamad Medical Corporation, Doha, QAT; 2 Medical Education Department, Hamad Medical Corporation, Doha, QAT; 3 Medical Education Department, Hamad General Hospital, Doha, QAT; 4 Nursing and Midwifery Research Department, Hamad Medical Corporation, Doha, QAT

**Keywords:** basal ganglia, calcium metabolism, cerebral cortex, fahr’s disease, neurological disease

## Abstract

Fahr's syndrome is a complex neurological disorder characterized by abnormal calcium deposition in the brain, leading to atrophy of the cerebral cortex, white matter, and basal ganglia. This case study examines a 28-year-old male patient to illustrate the remarkable clinical heterogeneity of the condition and its diagnostic challenges. Although rare, Fahr's syndrome can profoundly impact patients, presenting with a wide spectrum of symptoms, including motor deficits, cognitive decline, and diverse neurological manifestations. Current management remains predominantly supportive, aimed at controlling seizures and addressing neuropsychiatric complications. The presentation of this young male patient is particularly unusual, challenging conventional demographic assumptions and underscoring the necessity for individualized patient evaluation. Diagnosis relies heavily on neuroimaging, specifically computed tomography (CT) and contrast-enhanced magnetic resonance imaging (MRI), highlighting the critical role of appropriate imaging techniques given the disease/syndrome's variable presentations. This case not only confirms the extensive clinical variability of Fahr's syndrome but also contributes to the development of more personalized diagnostic and therapeutic approaches.

## Introduction

Fahr's disease/syndrome is a rare and complex neurological disorder driven by atypical calcium accumulation in the cerebral cortex, white matter tracts, and basal ganglia, ultimately leading to cerebral atrophy [1-3]. It can manifest through autosomal dominant or recessive inheritance, though a clear genetic basis is not always identified [1]. Typically presenting in the fourth or fifth decade of life, the disease is associated with a wide range of movement disorders and neuropsychiatric symptoms. These include cognitive deficits, dementia, seizures, psychosis, ataxia, and various motor impairments [2,3].

Due to its psychiatric presentation, Fahr's disease/syndrome is often misunderstood and misdiagnosed in psychiatric populations [1]. This underscores the critical need for further research and for documenting distinctive imaging findings to facilitate accurate diagnosis. We present an advanced radiological case of Fahr's syndrome in a young male exhibiting atypical clinical signs, including schizophrenia, microcephaly, and an aberrant gait.

Although genetic testing and family history were unavailable for this patient, the salient imaging data and symptomatology are instrumental in enhancing the recognition and understanding of Fahr's disease/syndrome across all age groups.

## Case presentation

Our patient is a 28-year-old Kenyan male who first presented to Hazm Mebaireek General Hospital (HMGH) (a member of Hamad Medical Corporation, Doha, Qatar) on 19 April 2025, presenting with right flank pain associated with vomiting. He was given analgesia by ambulance, and his pain subsided. He had a fever, but no urinary urgency or frequency. Had no past medical or surgical history. Abdominal examination revealed a soft, non-tender abdomen. He is not a smoker and does not consume alcohol. The bedside ultrasound showed no hydronephrosis. He was vitally stable and afebrile. He was diagnosed with a ureteric stone and was discharged on conservative treatment.

On 18 July 2025, he was brought to the emergency department (ED) by his relative, who reported that he had been terminated from his job one month prior after a conflict with a customer. Since then, he has stayed alone in his room, exhibiting abnormal behavior. For the last five days, he was deteriorating, suffering from restlessness, lack of sleep, poor food intake, observed to be talking to himself and reciting religious verses, and saying that the customer had him through black magic. He denied hallucination, delusions, or drug or alcohol consumption. On examination, he was vitally stable, conscious, alert, talking to himself, and reciting the Quran, with mild restlessness but no combative or agitated behavior. Equal air entry bilaterally on chest auscultation. Normal S1 and S2, and no added sounds on cardiac auscultation. His abdomen was soft, non-tender, and not distended. No skin rashes or lower limb edema. Denies any past psychiatric or family history. In the ED, the patient developed agitation and increased restlessness. The patient was then admitted to the hospital.

Investigation and diagnosis

**Figure 1:**
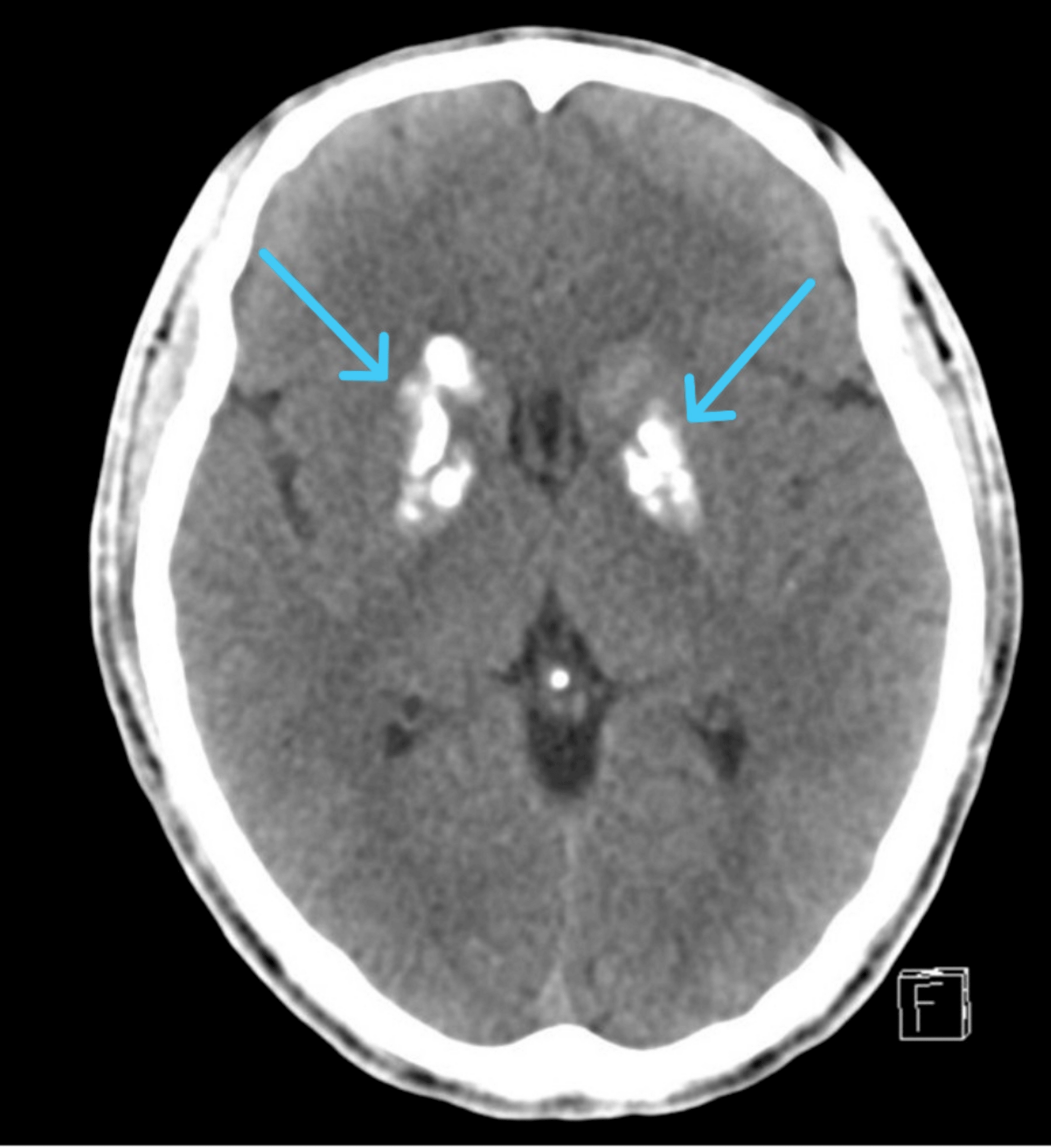
Brain CT scan showing intracranial calcifications in Fahr’s disease/syndrome. Dense bilateral calcifications are seen in the basal ganglia (blue arrows), with additional smaller calcifications in the subcortical frontal white matter, right corona radiata, and right dentate nucleus. These findings are characteristic of Fahr’s disease/syndrome, indicating abnormal calcium deposition across multiple neuroanatomical regions.

A CT head with contrast was performed on 20 July 2025, which showed no evidence of acute intracranial hemorrhage, dense bilateral basal ganglia calcifications, and small calcifications in the subcortical frontal white matter, right corona radiata, and right dentate nucleus (Figure 1). The findings suggested the possibility of Fahr’s syndrome/disease. Neurology and psychiatry teams were on board.

**Figure 2:**
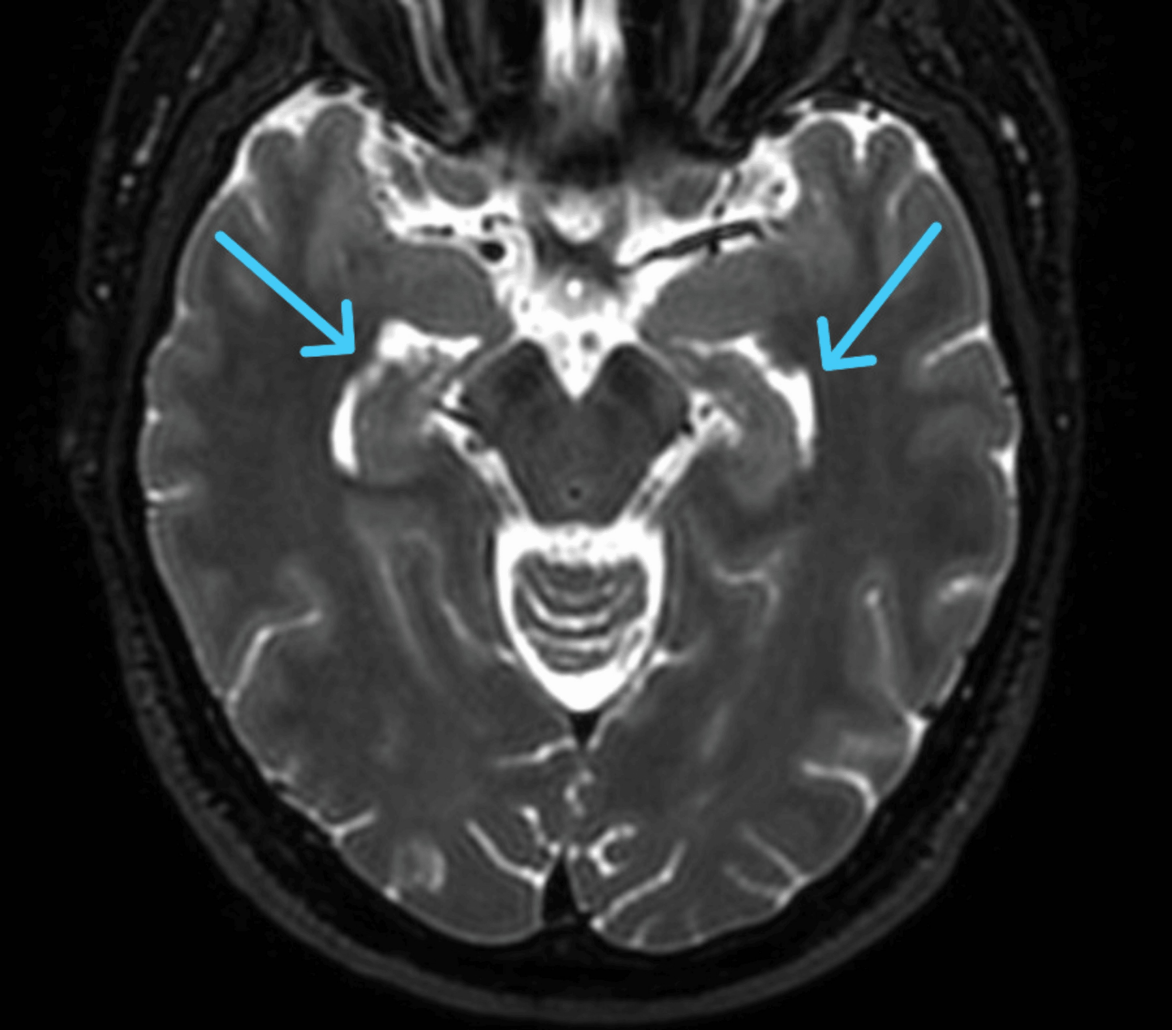
Brain MRI showing calcifications in Fahr’s syndrome. Dense calcification is evident in the basal ganglia, with lesser involvement of the cerebellar dentate nuclei (blue arrows). These imaging findings are suggestive of Fahr’s disease/syndrome, reflecting abnormal calcium deposition in deep gray matter structures.

An MRI head with contrast was performed on 22 July 2025 and confirmed dense calcification of the basal ganglia and, to a lesser extent, the cerebellar dentate nuclei (Figure 2). The imaging findings were consistent with Fahr’s disease/syndrome.

The laboratory profile is significant for an elevated PTH level in the setting of normocalcemia (Table 1). This pattern is consistent with secondary hyperparathyroidism. The most common cause for this is vitamin D deficiency, which was low in our patient (Table 1). Other potential causes include renal impairment or inadequate calcium intake. The chronic state of secondary hyperparathyroidism, by elevating the calcium-phosphate product, can predispose to ectopic calcification in susceptible tissues, such as the basal ganglia. This identifiable metabolic disturbance shifts the diagnosis from idiopathic Fahr's disease to symptomatic Fahr's syndrome.

**Table 1:** Relevant laboratory investigations.

Test	Patient's Result	Reference Range	Interpretation
Parathyroid Hormone (PTH)	66 pg/mL	15-65 pg/mL	Elevated
Serum Calcium	2.32 mmol/L	2.10-2.55 mmol/L	Normal
Serum Phosphorus	1.31 mmol/L	0.81-1.45 mmol/L	Normal (low-normal)
Serum Magnesium	0.82 mmol/L	0.70-1.00 mmol/L	Normal
Vitamin D (25-OH)	20 nmol/L	> 50 nmol/L (sufficient)	Deficient

Differential diagnosis

A structured differential for bilateral basal ganglia calcifications was considered. Fahr's syndrome was confirmed by the biochemical evidence of secondary hyperparathyroidism. The idiopathic form (Fahr's disease) was ruled out by the abnormal laboratory findings. Other causes, such as infection, mitochondrial disease, toxicity, or physiological aging, were deemed unlikely based on the clinical picture and investigation results.

Follow-up

A multidisciplinary team comprising neurology, psychiatry, and endocrinology specialists was involved in the patient’s care. The treatment approach focused on addressing both the neuropsychiatric presentation and the underlying metabolic abnormalities. Risperidone 2 mg orally once daily was initiated for two months to alleviate the psychotic symptoms and stabilize behavior. Concurrently, the patient was started on vitamin D supplementation at 50,000 IU weekly for two months to correct the deficiency contributing to secondary hyperparathyroidism. The patient was also referred to endocrinology for further diagnostic evaluation and long-term management. Additionally, psychoeducation was provided to the patient and his family, emphasizing the importance of medication adherence, understanding the nature of the condition, and ensuring regular follow-up to support optimal recovery and prevent relapse.

## Discussion

Fahr's disease/syndrome is an uncommon neurological condition defined by abnormal calcifications of the basal ganglia with no known cause. It typically manifests in patients aged 40-60. Patients are usually in good health in their youth and tend to develop this progressive neurodegenerative disease later in adulthood. Fahr’s disease/syndrome presentations vary, including neuropsychiatric features such as changes in behavior, concentration deficits, dementia, and depression. It can also present with signs such as dystonia and gait disturbance [4]. This case is rare, and it can often be confusing with other neurological and psychiatric diseases. Our 28-year-old patient presented with a new onset of abnormal behavior and delusional thinking with no past medical history of any psychiatric disorder. This early onset of Fahr's disease/syndrome underscores the importance of considering the condition in patients in their 20s, making it a valuable addition to the literature. Although multiple cases of Fahr’s disease/syndrome were reported with a past medical history of psychiatric illnesses such as schizophrenia, generalized anxiety disorder (GAD), and major depressive disorder (MDD), our patient did not have a clinical background of any psychiatric illness. Numerous cases of diagnosed Fahr’s disease/syndrome were observed with epileptic seizures [2,5,6]. A case of Fahr’s disease/syndrome mentioned in the literature presented with progressive signs of Parkinsonism, such as bradykinesia, in a young patient [7]. The patient did not exhibit any abnormal movements or signs resembling Parkinson's disease. A noticeable association between Fahr’s disease/syndrome and Ischemic stroke was also reported in the literature, with patients diagnosed with Fahr’s disease/syndrome who experienced signs such as facial numbness, hemiparesis, and dysarthria [7-9]. Fahr’s disease/syndrome is usually incidentally detected on imaging, as in our case. Physicians must consider the possibility of Fahr’s disease/syndrome to be the cause of the patient's clinical presentation, especially if other differentials are ruled out or if it is an atypical presentation.

It is important to follow up, as the early onset of Fahr's disease/syndrome progresses to other manifestations, typically movement disorders that may require management. Further documentation of Fahr's disease/syndrome is necessary, as it remains poorly understood.

## Conclusions

This case highlights an atypical and radiologically advanced presentation of Fahr’s syndrome in a young adult without prior psychiatric history, underscoring the importance of considering this rare neurodegenerative disorder even in early adulthood. The constellation of neuropsychiatric symptoms, including delusional thinking and social withdrawal, combined with striking basal ganglia calcifications on imaging, highlights the diagnostic value of neuroimaging in unexplained psychiatric and neurological presentations. Given the absence of genetic testing and the unclear family history, this case also highlights the importance of heightened clinical suspicion and multidisciplinary collaboration in diagnosing Fahr’s disease/syndrome. Early recognition is crucial, as progression may involve movement disorders and cognitive decline requiring long-term management. Further documentation of such atypical cases is essential to expand the clinical spectrum, improve diagnostic accuracy, and guide future research into the pathophysiology and management of Fahr’s disease/syndrome.
